# The lack of clinical value of peritoneal washing cytology in high risk patients undergoing risk-reducing salpingo-oophorectomy: a retrospective study and review

**DOI:** 10.1186/s12885-015-2011-5

**Published:** 2016-01-14

**Authors:** F. Blok, E. M. Roes, G. J. L. H. van Leenders, H. J. van Beekhuizen

**Affiliations:** Bachelor of Medicine at the Erasmus University Rotterdam, Rotterdam, The Netherlands; Gynecologic Oncologist at Erasmus Medical Centre Cancer Institute, Rotterdam, The Netherlands; Pathologist at Erasmus Medical Centre Rotterdam, Rotterdam, The Netherlands; Department of Obstetrics and Gynecology, Erasmus Medical Centre Cancer Institute, DHD-420, PO box 5201, 3008 AE Rotterdam, The Netherlands

**Keywords:** BRCA1, BRCA2, Risk reducing surgery, Peritoneal washing cytology, Primary peritoneal cancer

## Abstract

**Background:**

To assess the clinical value of peritoneal washing cytology (PWC) in women with BRCA1 or BRCA2 mutations and women from a family with hereditary breast and/or ovarian cancer (HBOC) undergoing risk-reducing salpingo-oophorectomy (RRSO) in detecting primary peritoneal cancer (PPC) or occult ovarian/fallopian tube cancer.

**Methods:**

A retrospective study of patients with known BRCA1 or BRCA2 mutation or HBOC who underwent RRSO at the Erasmus Medical Centre, Rotterdam, The Netherlands between January 2000–2014. Patients with an elevated risk of malignancy prior to the procedure were excluded from primary analysis (elevated CA-125, an ovarian mass, abdominal pain or another gynecological malignancy). A review of the literature was conducted.

**Results:**

Of the 471 patients who underwent RRSO, a total of 267 cytology samples were available for analysis. Four samples showed malignant cells, all four patients were diagnosed with ovarian and/or fallopian tube cancer at histologic examination. A fifth patient, of whom no cytology sample was obtained during RRSO, developed primary peritoneal cancer 80 months post RRSO.

**Conclusions:**

This study failed to show that cytology is of value during RRSO in detecting primary peritoneal cancer, however 36 % of patients with concomitant ovarian or fallopian tube cancer had positive cytology. Therefore, the routine sampling of peritoneal washings during RRSO is not found to be useful to detect subsequent PPC.

## Background

Peritoneal washing cytology (PWC) has been used for years in gynecological surgery to detect metastasis and to stage malignant gynecologic cancers. It has minimal risk for the patient and may be useful in the detection of early dissemination of cancer. Although PWC can be done easily while performing laparoscopy, routine testing in patients with presumed benign disease has been discouraged to save money and to avoid distressing false positive test results [1, 2]. In patients diagnosed with cancer, such as ovarian, fallopian tube or endometrial cancer, the PWC outcome gives additional information about the prognosis [3] and has influence on postoperative staging of ovarian and fallopian tube cancer [4, 5]. The main reason for performing PWC in patients undergoing risk-reducing salpingo-oopherectomy (RRSO), is to detect early ovarian and fallopian tube cancer which may be too small to detect by histology examination of tubes and ovaries, and to detect primary peritoneal carcinoma (PPC).

The main indications for RRSO are risk-reducing surgery in women at risk of developing ovarian or fallopian tube cancer such as BRCA1 or BRCA2 germline mutations or hereditary (breast) and ovarian cancer (HBOC) [1]. Women affected by a BRCA1 or BRCA2 germline mutation have a 20–40 % [6, 7] and 15–25 % [6] lifetime risk, respectively, of developing a gynecologic cancer. Percentages of finding unsuspected carcinomas at RRSO vary from 6 to 17 % [8, 9] there is also a 5–6 % chance of finding a serous tubal intraepithelial carcinoma (STIC) at RRSO, which is thought to give rise to high grade serous carcinoma [10]. RRSO is a highly protective procedure against the development of ovarian- and fallopian tube cancers in this patient group (hazard ratio of RRSO for the development of breast and BRCA-related gynecologic cancer: 0.21, 95 % CI 0.07–0.62) [11], however a 1–6 % lifetime risk of developing PPC still exists after this procedure [12]. HBOC patients do not carry a BRCA1 or BRCA2 mutation but have an increased risk of developing breast and ovarian cancer [13].

PWC appears to be a harmless and effortless technique to detect malignant gynecologic cancers but it is not completely clear yet how to interpret the findings. A false positive PWC in women will cause unnecessary anxiety and possibly leads to unnecessary diagnostic testing. Conversely, a positive test result in women who have (subclinical) PPC, PWC may lead to earlier diagnosis and possibly improve the prognosis. Studies on PPC have shown that the median survival is 23.5 (95 % CI, 18.6–39.8) [14] to 42 (95 % CI 22–62) months [15], but the advantage in survival for early diagnosis of PPC is not known.

In this retrospective study of women with a known BRCA mutation or a HBOC family undergoing RRSO, first we investigated the clinical value of malignant PWC in detecting ovarian and fallopian tube cancer and the early detection of PPC. Second, we study the correlation between PWC and tuba/ovarian malignancies and PPC in the setting of RRSO.

## Methods

This is a retrospective study of patients taken from data in the electronic health records of the Department of Gynecologic Oncology of the Erasmus MC Cancer Institute Rotterdam, The Netherlands. RRSO is performed in patients with > 10 % risk of developing ovarian cancer. Electronic health records were used to select patients who underwent RRSO between January 2000 and January 2014. The criteria for HBOC were followed in accordance to our national guidelines [16]. To be certain no patients were missed during the selection, the local electronic pathology registry was searched, using the terms ‘ovary’ and ‘tube’ and all selected patients were crosschecked in the FAMOND database (a database including all patients with a high risk of developing ovarian and/or breast cancer that have been treated in the Erasmus MC Cancer Institute Rotterdam). Patients excluded from this study include those whose salpingo-oophorectomy (SO) was indicated because of pre-operative medical complaints or suspicious abnormalities at transvaginal ultrasound (TVUS). Also patients undergoing SO because of elevated CA-125 by follow-up were excluded. Because CA-125 measurements were obtained routinely in patients with a known BRCA mutation, patients with a retrospectively elevated CA-125 post RRSO were not excluded. This because the study aims to include all patients with an absolute prophylactic indication for RRSO.

Since 2007, PWC has been routinely performed during RRSO according to our hospital protocol. Surgery procedures during RRSO included PWC, removing the ovaries, fallopian tubes and mesosalpinx. Laparoscopy is the standard procedure for performing a RRSO. As soon as the peritoneal cavity is filled with CO_2_ and the instruments are placed, then incidental free fluid is aspirated. If free fluid is not present, 10-100 ml saline (0.9 % natriumchloride solution) is introduced to lavage the peritoneal cavity. Displacement of cervical, endometrial and endosalpingeal tissues can occur and potentially dislodge cells, thereby contaminating cytology fluids. Therefore, PWC is performed immediately after opening the peritoneal cavity, reducing the likelihood of dislodging cells [17]. The surgery report noted whether free fluids or ascites was present in the peritoneal cavity and if present, a PWC sample and/or (if available) ascites was collected before manipulation of pelvic organs. In our hospital, cytology and histology samples are sent to two different laboratories, allowing for a double-blinded study design. Pathologists do not consult each other, except when results are inconclusive. The fluid was centrifuged in the laboratory and stained using the Papanicolaou and Giemsa method. Samples were categorized according to the following categories, no analysis possible because of poor quality of the sample, benign, atypical, suspicious for malignancy or malignant cells. The presence of psammoma bodies and endosalpingiosis was also noted, to prevent false positive malignant findings [18]. Histology of the RRSO specimens was assessed using microscopic examination, using the standardized SEE-FIM protocol for the evaluation of all specimens obtained from 2006 to present [19]. This protocol maximizes the proportion of the fallopian tube mucosa. An increase of approximately 60 % surface area of the fimbria is obtained as compared to the conventional serial cross-sectioning [19]. The findings of the histology were classified in the following categories: no malignancy, benign cyst, cystic teratoma, adeno(fibro)ma, borderline tumor or malignancy of the ovaries and/or fallopian tubes.

Permission for this retrospective study was granted by the medical ethical committee of our hospital, according to the regulations (Medical ethical committee number: MEC-2015-036) [20].

Data were collected and analyzed using SPSS version 21. The results were described using descriptive statistical methods (mean ± SD). Results were analyzed using the Student *T* test for continuous data and the Chi square test or Fischer’s exact test for binary data, depending on sample size of the subgroup. A p-value <0.05 was considered to be significant. Calculation of sensitivity, specificity, number-needed-to-treat (NNT), positive and negative predictive value (PPV and NPV) and the positive and negative likelihood ratio (LR) were planned. Furthermore, the risk factors of developing PPC were planned to be analyzed using logistic regression. A Kaplan Meier survival plot was planned for patients with positive and negative PWC. The difference between the sensitivity and specificity of ascites versus PWC samples was also planned to be calculated, along with the difference in malignant outcome of histologic examination of the RRSO specimen.

This study also conducted a literature search using PubMed. The relevant articles are discussed in the discussion section of this article.

## Results

**Fig. 1:**
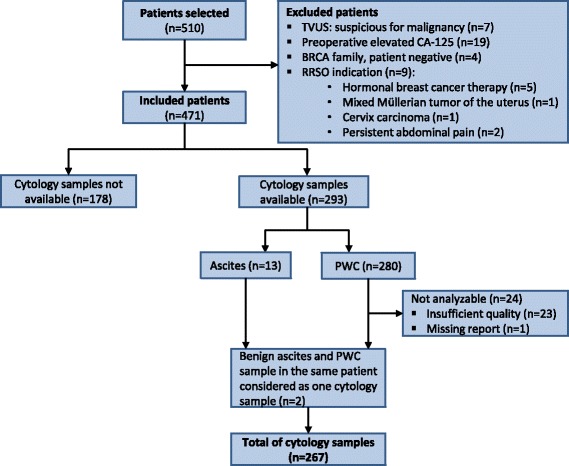
Flowchart of excluded patients and cytology samples

Five hundred and ten patients were included during the study period. 39 patients were excluded due to indications for (RR)SO which were not prophylactic (see Fig. 1). Seven of the included patients already underwent an ovariectomy between 1995 and 1998, but received an additional salpingectomy between January 2000 and January 2014. Of the remaining 471 patients, 288 had BRCA1 mutations, of those one patient had a 50 % chance of having a BRCA1 mutation, 126 had BRCA2 mutations, 2 had both BRCA1 and BRCA2 mutations. Of the 55 patients in the HBOC group, 12 did not meet all the criteria for HBOC and in four patients information about family history was not available. The median age of patients at the time of RRSO was 48 years (range, 33–78 years). See Table 1 for descriptive statistics for this group.

**Table 1:** Descriptive statistics of the total group of patients who underwent RRSO (*n* = 471)

	Number	Percentage	Range
Age (median, year)	48	%	33–78
*Mutations*			
-BRCA1	288	61.1	
-BRCA2	126	26.8	
-BRCA1 and BRCA2	2	0.4	
-HBOC	55	11.7	
*CA-125 preoperative (n)*	466		
-median (U/ml)	14.0		4.0–93.0
-elevated CA125 (>35 U/ml) (n)	10	2.1	
*Surgical technique (n)*	471		
-laparoscopy	430	91.3	
-laparotomy	37	7.9	
-vaginal	3	0.6	
-combined with supravaginal uterus extirpation	1	0.2	
*Documented complications (n)*	444	94.3	
-no complications during surgery and/or postoperatively	416	93.7	
-complications	28	5.9	
-converted to laparotomy because of adhesions	10		
-hematoma	10		
-iatrogenic injury (intestine/ureter)	3		
-incomplete removal of the ovaries	1		
-not possible to remove ovaries because of adhesions	1		
-other	3		
*Menopausal status (n)*	447		
-postmenopausal	187	41.8	
-because of hormonal breast cancer treatment	32	17.1	
-because of previous ovariectomy	8	4.3	
-premenopausal	241	53.9	
-perimenopausal	19	4.3	
-because of hormonal breast cancer treatment	10	52.6	
*HRT in premenopausal women (n)*	217		
-postoperative usage of HRT	97	45.3	
*Follow up (median, mo) (n = 375)*	55.8		0.6–169.0
-deceased in FU	19	5.1	
-age (median, year)	55.5		43.1–72.0
-development of breast cancer in FU	57	12.1	
-development of PPC in FU	1	0.2	
-development of other malignancy	22	4.7	
*Previous breast cancer (n)*	195	41.4	
-age first breast cancer (median, year)	41.0		24.7–64.0
*Cytology*			
PWC sample not available	191	40.6	
PWC sample available	280	59.4	
-PWC analyzable	257	91.8	
-PWC malignant	4	1.6	
-PWC atypical	1	0.4	
Ascites sample reported	21	4.5	
-Ascites sample available for analysis	13	61.9	
-ascites malignant	0	0	
-ascites atypical	0	0	
*Histology RRSO specimen*			
Ovarian (n)	469		
-benign	399	85.1	
-primary serous adenocarcinoma	4	0.9	
-granulosa cell tumor	1	0.2	
-borderline tumor	1	0.2	
-stromal hyperplasia	2	0.4	
-mature cystic teratoma (benign)	4	0.9	
-cyst (serous/mucinous/simple/endometriosis/Theca lutein/corpus luteum)	43	9.2	
-cystadenoma/cystadenofibroma	15	3.2	
Fallopian tube (n)	470		
-benign	433	92.1	
-adenocarcinoma	5	0.6	
-serous large cell carcinoma	1	0.2	
-benign mesothelial hyperplasia	1	0.2	
-epithelial dysplasia	1	0.2	
-cystadenoma/cystadenofibroma	9	1.9	
-endometriosis	7	1.5	
-endosalpingiosis	13	2.8	

In one patient both ovaries remained in situ because of multiple adhesions discovered during the RRSO procedure. Cytology samples were not available for this patient. PWC was available for 280 patients. The presence or absence of ascites was reported in 21 surgical reports, 13 ascites samples were available for analysis. PWC samples were obtained in 48 patients (24.6 %) of the RRSO procedures between January 2000 to December 2006 and from January 2007 to December 2013 in 232 patients (84.1 %). Further analysis did not show a significant difference in the number of malignant PWC samples between these periods (*p* = 0.51). The quality of 23 PWC samples were too poor for analysis and the report of one PWC sample was missing in the electronic patient file. Malignant cells were detected in four of the remaining 256 PWC samples. No atypical or malignant cells were found in the ascites samples. Since some of the samples were inadequate, PWC and ascites samples were analyzed as one group, resulting in a total of 267 samples. In two patients both ascites and PWC samples were reported in the surgical report. Because all of these samples were benign, PWC and ascites samples were considered as one sample for each patient (see Fig. 1). Malignancy in cytology samples was significantly related to malignancy in histology samples in the ovaries and fallopian tubes (*p* < 0.001). The results of these findings are represented in Table 2. In total 11 patients had fallopian tube or ovarian cancer, all primarily diagnosed with the RRSO specimen. In 4 (36 %) of these 11 patients the PWC samples were positive.

**Table 2:** Results of peritoneal washing cytology in RRSO (*n* = 267)

	Malignant cytology	Benign cytology	*Total*
Malignant histopathology	4	7	*11*
Benign histopathology	0	256	*256*
*Total*	*4*	*263*	*267*

*P* < 0.001, using the Fisher’s exact test

Surgery reports were checked for uterine manipulator factors prior to the collection of cytology samples which could have caused potential dislodgement of cells, resulting in contamination of the samples. In surgery reports of four patients with malignant PWC, only one mentioned the timing of PWC sampling. The sampling had taken place after insufflating the abdominal cavity and inspection of the abdominal cavity, uterus and fallopian tubes. In one of the patients in which the timing of PWC was not mentioned, the laparoscopic procedure was converted to open surgery because of adhesions.

Of the four patients with malignant PWC, two patients had fallopian tube cancer (FIGO stage IIC and IIIA) and two ovarian cancer (FIGO stage IIC and IIIC). All histology samples were conclusive and none of the malignancies was detected through the malignant PWC sample. After a median FU of 58 months (range, 46–111) no evidence of disease was found in three of the four patients. The fourth patient, with progressive ovarian carcinoma, was lost in follow up.

One sample in this study was reported as atypical PWC and although malignant cells were reported in the first cytology report, no adenocarcinoma was revealed in the RRSO specimen. Both the RRSO specimen and the PWC sample were reassessed. Pathologists concluded there was no malignancy in the RRSO specimen and they attributed the atypical cells in the PWC to endometriosis. This conclusion was confirmed by reference pathologists at the request of second opinion. This patient was followed up for 39 months without having developed malignancy and then was lost to FU after that. Only one patient, aged 60 years did develop PPC 80 months post RRSO, although no malignancy was found at RRSO even though no PWC or ascites samples were collected. The diagnosis of PPC was confirmed by biopsies and the patient was treated with induction chemotherapy, interval debulking surgery and adjuvant chemotherapy. To date, no progression or recurrence has been noted during the 34 months FU at our hospital. In seven other cases ovarian or fallopian tube malignancy was revealed by histology, but cytology samples did not show malignant cells. The histology of the ovarian and tubal cancers are depicted in Table 1.

## Discussion

Four malignant cytology samples were found in this 14-year retrospective study of women who underwent RRSO, which included cytology samples of 471 women with BRCA1 and/or BRCA2 mutation or a strongly positive family history (HBOC). Two of these patients had ovarian cancer and two patients had fallopian tube cancer with metastases in the ovaries at histologic examination. In follow-up, no PPC developed in patients who underwent PWC sampling. Only one patient (without PWC obtained at the time of the RRSO) developed PPC at 80 months follow-up, which was confirmed by biopsy. In seven other patients a malignancy was found at histology, but PWC did not reveal any malignant cells. There were significantly more malignant cytology samples in the group of patients in whom the RRSO specimen revealed malignancy at histopathologic examination (*p* < 0.001). Because histologic and cytological examinations are analysed in independant laboratories, it is certain that no malignant outcomes at histology were found because of malignant outcomes at cytology. One PWC sample showed atypical cells. Studies have shown that reactive mesothelial cells, endometriosis and endosalpingiosis could give false positive results [18]. Dislodgement of cells could contaminate samples. Of four patients with malignant PWC, only one surgery report mentioned the timing of PWC sampling, which occurred immediately following the inspection of the abdominal cavity, uterus and fallopian tubes. Another surgery report of a patient with malignant PWC did not mention the timing of PWC, but noted that the procedure was converted because of adhesions. Difficulties introducing instruments and CO_2_ insufflation prior to conversion could have caused contamination of the sample.

Literature shows that while PWC is able to detect malignant cells in patients undergoing RRSO [1, 17, 21–24], but the numbers of positive PWC samples in these studies are minimal and the added value of performing a PWC in detecting PPC remains uncertain. These studies are depicted in Table 3. PWC was performed in 836 patients and malignancy was demonstrated in 15 (1.8 %) of those patients, 14 (93 %) were found to have concomitant ovarian/fallopian tube cancer. Two of these 14 patients (13.0 %) had concomitant ovarian/fallopian tube cancer with peritoneal metastases. There were no patients diagnosed with subsequent PPC. Ovarian and/or fallopian tube cancer were found in 37 patients, of whom 14 patients (37.8 %) had a positive PWC sample. In one patient (0.12 %) with a malignant PWC histologic examination of the RRSO specimen did not reveal any malignancy [22]. In total, 569 PWC samples were collected. Eleven of these samples were positive for malignancy and one of these PWC samples was false positive when looking at ovarian and fallopian tube cancer (see Table 3). In most studies, the SEE-FIM protocol- that examines the fimbriated end more extensively- was not used. This could have caused false negative findings during histological examination of the RRSO specimen. This could provide an explanation for the patient in the study of Colgan et al. [22] with a malignant PWC, but no malignancy in the RRSO specimen. Additional information provided by Leunen et al. [23] none of the patients developed PPC to date. Unfortunately, the rest of the authors of the studies listed in Table 3 were not willing or able to provide more information about the recent FU of their patients. It is possible that due to publication bias studies with high incidence of malignant PWC are published.

**Table 3:** Summary of studies (including present study) researching PWC in RRSO among women with a BRCA mutation or a pedigree analysis that showed a chance >50 %

Study	Number	Median age (yr) (range)	Median FU (mo)	PWC sample available (n)	Malignant PWC (n)	Malignant PWC associated w/ ovarian/fallopian tube cancer (n)	Benign PWC associated w/ovarian/fallopian tube cancer (n)	Malignant PWC and PPC during RRSO	Malignant PWC and PPC in FU (n)	Malignant PWC not associated w/ovarian/fallopian tube cancer (n)	Median age women w/malignant PWC (yr)	Median FU women w/malignant PWC (mo)
Colgan et al. [12]	35	NA	NA	35	3^a^	2^a^	1	0	0	1^a^	NA	NA
Leunen et al. [14]	51	45^g^	25	28	0	-	0	-	-	-	-	-
Eitan et al. [13]^b^	130	48 (33–78)	20	117	0	-	0	-	-	-	-	-
Haldar et al. [1]	113	52 (35–78)	34	110	2	2^c^	0	0^c^	0^c^	0	NA	NA
Landon et al. [15]	116^d^	NA	NA	116^d^	0^d^	0^d^	9	0	-	-	NA	NA
Chen et al. [11]	163	NA	NA	163	6	6	6	0	0^e^	0	NA	NA
Present study Blok et al.	471	48	56	267	4	4	7	0	0^f^	0	47.6 (SD, 8.5)	58
Total	1079			836	15	14	23	0	0	1		

*Abbreviation*: *NA* not available, *SD* standard deviation

^a^) In one of three patients with a malignant PWC, histopathology did not show any malignancy. No malignancy was detected at second-look laparotomy in peritoneal biopsies and PWC. The patient was treated with chemotherapy and there is no evidence of disease 10 months FU. ^b)^ This study also included patients for whom RRSO was indicated because of a personal history of breast cancer. ^c^) The authors report that in total two women had a positive PWC, histological evidence of ovarian and/or fallopian tube cancer and histological evidence of PPC at time of RRSO. The authors of this article interpret these results as ovarian and/or fallopian tube cancer with peritoneal metastasis. ^d^) One patient had a malignant PWC, but no malignancy at histopathology (confirmed by four cytopathologists). The patient was treated with chemotherapy for presumed PPC. At second-look laparotomy, peritoneal biopsies and PWC did not reveal any malignancy. The patient had no evidence of disease 118 months FU. Because of the doubtful diagnosis of PPC, this sample was not taken into account in this article. ^e^) One patient with malignant PWC and histopathological evidence of ovarian and/or fallopian tube cancer, developed PPC at 47 months FU. Because of the previous ovarian and/or fallopian tube cancer in this patient, we would prefer to call this finding recurrence of disease instead of PPC. Another patient with benign PWC developed PPC or recurrence of ovarian carcinoma at 81 months FU. ^f^) In this study, one patient developed PPC 80 months FU. At RRSO, there was no evidence of malignancy at histopathologic examination. There was no PWC sample available.

^g^) mean age

Since 2007 the RRSO protocol in our hospital states that PWC should be collected routinely. In our study, we notice that PWC was collected more frequently in the last seven years of the study period then when compared with the first seven years. This may also indicate that in the first half of the study period the indication for obtaining PWC was different from the second half, but no difference in the amount of malignant PWC samples was found between the two 7-year periods (*p* = 0.51). This study included all patients who underwent RRSO up to January 2014, which caused a shorter follow up time for patients who were included more recently. Though the overall median FU period is relatively long (median, 55.8 months (range, 0.6–169.0)). This study excluded all patients who underwent RRSO because of suspected malignancy. A further, more extended statistical analysis was not possible with the low incidence of PPC.

This study, which included the largest number of cytology samples of patients undergoing RRSO to date, failed to show that cytology is of value during RRSO for early detection of PPC. Malignant cytology samples were extremely rare (1.5 %) and if present, malignancy was found in the ovaries and/or fallopian tubes. In literature only two patients (out of 569 PWC samples) with malignant PWC who had ovarian/fallopian tube cancer with peritoneal metastasis were reported. Including our study, two of 836 (0.24 %) patients with positive PWC developed concomitant PPC. No patients developed subsequent PPC. Furthermore, malignant PWC samples failed to add any value to histopathological examination in detecting ovarian and/or fallopian tube cancer when using the SEE-FIM protocol.

Although the collection of cytology samples is relatively simple, it is costly. Our pathology department charges an amount of €77.09 (USD 83.53) per cytology sample, which includes all technical costs and pathologists honorary.

## Conclusions

We recommend that PWC should not be practiced routinely at RRSO in high risk patients, preventing unnecessary testing and use of resources. In case of malignancy at histopathology we suggest to perform PWC at second-look staging surgery.

## Abbreviations

FU: Follow up
HBOC: Hereditary breast and/or ovarian cancer
LR: Likelihood ratio
NNT: Number needed to treat
NPV: Negative predictive value
PPC: Primary peritoneal cancer
PPV: Positive predictive value
PWC: Peritoneal washing cytology
RRSO: Risk-reducing salpingo-oophorectomy
SO: Salpingo-oophorectomy
STIC: Serous tubal intraepithelial carcinoma
TVUS: Transvaginal ultrasound
